# Differential Effects of DHA- and EPA-Rich Oils on Sleep in Healthy Young Adults: A Randomized Controlled Trial

**DOI:** 10.3390/nu13010248

**Published:** 2021-01-16

**Authors:** Michael J. Patan, David O. Kennedy, Cathrine Husberg, Svein Olaf Hustvedt, Philip C. Calder, Benita Middleton, Julie Khan, Joanne Forster, Philippa A. Jackson

**Affiliations:** 1 Brain, Performance and Nutrition Research Centre, Health and Life Sciences, Northumbria University, Newcastle upon Tyne NE1 8ST, UK; Michael.j.Patan@northumbria.ac.uk (M.J.P.); david.kennedy@northumbria.ac.uk (D.O.K.); julie.khan@northumbria.ac.uk (J.K.); jo.forster@northumbria.ac.uk (J.F.); 2 BASF AS, NO-0283 Oslo, Norway; Cathrine.Husberg@basf.com (C.H.); Svein.Olaf.Hustvedt@basf.com (S.O.H.); 3 Human Development and Health, Faculty of Medicine, University of Southampton, Southampton SO16 6YD, UK; P.C.Calder@soton.ac.uk; 4 National Institute of Health Research Southampton Biomedical Research Centre, University Hospital Southampton National Health Service Foundation Trust and University of Southampton, Southampton SO16 6YD, UK; 5 Faculty of Health & Medical Sciences, University of Surrey, Guildford GU2 7XH, UK; b.middleton@surrey.ac.uk

**Keywords:** docosahexaenoic acid, eicosapentaenoic acid, omega-3, sleep, actigraphy, SMEDS

## Abstract

Emerging evidence suggests that adequate intake of omega-3 polyunsaturated fatty acids (*n*-3 PUFAs), which include docosahexaenoic acid (DHA) and eicosapentaenoic acid (EPA), might be associated with better sleep quality. N-3 PUFAs, which must be acquired from dietary sources, are typically consumed at suboptimal levels in Western diets. Therefore, the current placebo-controlled, double-blind, randomized trial, investigated the effects of an oil rich in either DHA or EPA on sleep quality in healthy adults who habitually consumed low amounts of oily fish. Eighty-four participants aged 25–49 years completed the 26-week intervention trial. Compared to placebo, improvements in actigraphy sleep efficiency (*p* = 0.030) and latency (*p* = 0.026) were observed following the DHA-rich oil. However, these participants also reported feeling less energetic compared to the placebo (*p* = 0.041), and less rested (*p* = 0.017), and there was a trend towards feeling less ready to perform (*p* = 0.075) than those given EPA-rich oil. A trend towards improved sleep efficiency was identified in the EPA-rich group compared to placebo (*p* = 0.087), along with a significant decrease in both total time in bed (*p* = 0.032) and total sleep time (*p* = 0.019) compared to the DHA-rich oil. No significant effects of either treatment were identified for urinary excretion of the major melatonin metabolite 6-sulfatoxymelatonin. This study was the first to demonstrate some positive effects of dietary supplementation with *n*-3 PUFAs in healthy adult normal sleepers, and provides novel evidence showing the differential effects of *n*-3 PUFA supplements rich in either DHA or EPA. Further investigation into the mechanisms underpinning these observations including the effects of *n*-3 PUFAs on sleep architecture are required.

## 1. Introduction

The relationship between diet, which includes both specific dietary components and eating behaviors, and sleep quality and duration, is complex and bi-directional in nature [1]. Whilst emerging evidence suggests that obesity and following a high-fat, high-carbohydrate diet might be detrimental to sleep, conversely, improving micronutrient status (e.g., iron, zinc, magnesium, vitamin D, vitamin B12) and consumption of particular whole foods (e.g., milk, kiwi, tart cherries, oily fish) might have beneficial effects [2,3]. Oily fish is rich in the omega-3 polyunsaturated fatty acids (*n*-3 PUFAs) docosahexaenoic acid (DHA) and eicosapentaenoic acid (EPA), which are not easily produced endogenously in humans and so must be acquired from the diet. EPA and DHA are incorporated into the membranes of cells throughout the body, and DHA is particularly enriched in the brain. As such, adequate intake of these important fatty acids ensures proper functioning across multiple systems. Indeed, low levels of circulating *n*-3 PUFAs were observed in a wide range of psychopathologies, including attention deficit hyperactivity disorder, major depression, and Alzheimer’s disease [4].

Converging evidence suggests *n*-3 PUFAs are also important for sleep. Studies of dietary deficiency of *n*-3 PUFAs in experimental animals revealed a number of mechanisms through which DHA might specifically affect sleep regulation, including impaired functioning of the superchiasmatic nuclei [5], altered melatonin release [6], and disruption to endocannabinoid signaling [7]. With regard to behavioral effects, *n*-3 PUFA deficiency in rodents results in disorganized sleep patterns [6], an observation that was paralleled in children during a period of total parenteral nutrition devoid of lipids [8]. In humans, higher maternal levels of DHA appear to be linked to more mature infant sleep patterns [9,10]. Further, lower levels of DHA and a lower ratio of DHA to arachidonic acid (an n-6 PUFA) were negatively associated with parent ratings of children’s total sleep disturbance [11]. Likewise, the concentration of *n*-3 PUFAs in adipose tissue of patients with obesity suffering from sleep apnea was positively associated with sleep efficiency and minutes spent in slow wave sleep and rapid eye movement (REM) sleep [12].

Results from an exploratory pilot trial in children (*n* = 43, age 7–9 years) indicated that dietary supplementation with DHA might improve objectively measured sleep [11]. However, more data are needed. In addition, to our knowledge no studies evaluated the effects of EPA, which might also be relevant, given the previously observed effects of *n*-3 PUFAs on serotonin release [13] and the production of prostaglandins [14]; prostaglandin D2, in particular, is a potent somnogen known to mediate the sleep/wake cycle [15]. Therefore, the present study investigated the effects of 26 weeks’ supplementation with oils rich in either DHA or EPA on subjective and objective sleep quality in healthy, adult, low consumers of oily fish.

## 2. Materials and Methods

### 2.1. Study Design

This study employed a randomized, placebo-controlled, double-blind, parallel groups design. Participants were randomly assigned to receive one of three treatments for 26 weeks (placebo, DHA-rich oil, EPA-rich oil).

### 2.2. Participants

Prior to screening, all participants received information about the study and its procedures and signed an informed consent form. Participants were aged between 25–49 years and had to pass a physical/lifestyle screening to demonstrate they were in good health. Participants self-reported consumption of oily fish of less than once per week, measured via a DHA food frequency questionnaire [16]. Having good health was identified as being a non-smoker, free from prescription, herbal, illicit, or recreational drugs (females taking the contraceptive pill were included), free from major illnesses, having a blood pressure lower than 159/99 mmHg and a BMI between 18.5 and 35 kg/m^2^. All participants were recruited via posters, adverts placed on social media websites, or emails sent out to university staff and students, and were either students or staff attending/working at Northumbria University or individuals living in the Newcastle-upon-Tyne surrounding area.

**Figure 1:**
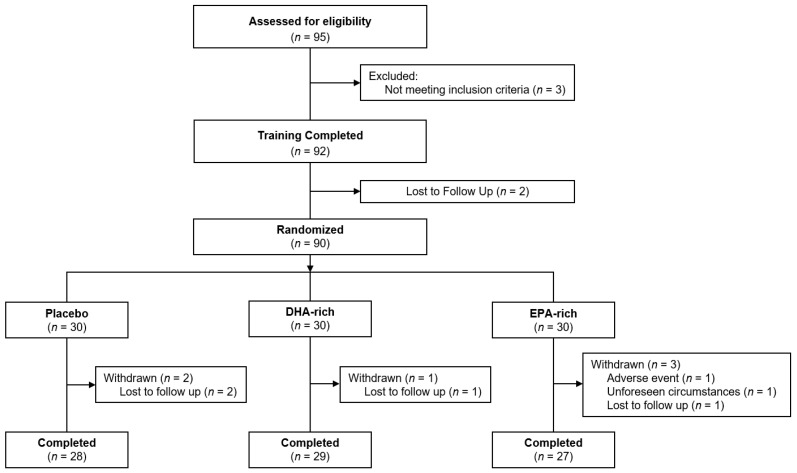
Participant disposition through the trial. Figure depicts the disposition of participants throughout the study, culminating in *n* = 84 of the 90 who were randomized.

Ninety-five males and females were screened for eligibility, 90 were enrolled into the study and 84 completed all study requirements. Of the six participants that did not complete the study, four were lost to follow-up after completion of the baseline testing visit, one withdrew consent and one was advised to stop adhering to the consumption of supplements due to reports of minor adverse events. Participant disposition throughout the trial is displayed in Figure 1, demographic data are shown in Table 1 and outcomes from the DHA food frequency questionnaire are shown in Table 2.

### 2.3. Sample Size

Sample size was calculated based on a medium effect size reported by Montgomery et al. [11] for total minutes asleep measured via actigraphy, following 16 weeks’ supplementation with DHA. Given this effect size, an a priori calculation of the size of sample required in order to detect a significant difference between the groups given 80% power and an alpha level of 0.05, was 30 participants per treatment arm, inclusive of a 10% anticipated dropout rate. Power calculations were made using GPower 3.1.3.

### 2.4. Randomization

Treatment group was assigned randomly, according to a randomization schedule produced using the website www.randomization.com. To ensure blinding was maintained throughout the study, a third party within the same university created the randomization schedule and coded treatments before the treatments and randomization schedule were delivered to the research team. Capsules were provided in opaque containers. Therefore, both the research team and participants were blind as to which participants received which treatment, until after data analysis was complete.

### 2.5. Treatment

All treatment capsules were supplied by BASF AS. Treatment was provided as three 1 g capsules. The DHA-rich capsules provided 900 mg DHA/d and 270 mg EPA/d (Accelon™ DHA EE EU capsules), the EPA-rich capsules provided 360 mg DHA/d and 900 mg EPA/d (Accelon™ EPA EE EU capsules) and the placebo capsules contained 1 g refined olive oil. Each capsule of Accelon High DHA contained 600 mg oil with at least 420 mg omega-3 fatty acid EEs, including EPA, DHA, C18:3 *n*-3, C18:4 *n*-3, C20:4 *n*-3, C21:5 *n*-3, and C22:5 *n*-3. The amount of DHA was at least 300 mg and EPA at least 90 mg per capsule. Similarly, each capsule of Accelon High EPA contained 600 mg oil with at least 450 mg omega-3 fatty acid EEs, including the same fatty acids as above. The amount of DHA was at least 120 mg and EPA was at least 300 mg per capsule. The additional capsule fill, 400 mg/capsule for both formulations, was food additives (permitted for use in food supplements). The active treatments using the Accelon™ technology also contained a proprietary mixture of surfactants and co-solvents. When exposed to the contents of the stomach, these ingredients are designed to spontaneously emulsify the oils, forming microdroplets. Known as a self-microemulsifying delivery system (SMEDS), this approach improves the absorption of the *n*-3 PUFAs contained within the treatments [17]. Participants were instructed to take their capsules with a glass of water at their usual bedtime. Placebo and treatment capsules were identical in size and shape, and similar in appearance.

### 2.6. Procedure

All study visits took place at Northumbria University’s Brain, Performance, and Nutrition Research Centre (BPNRC). Potential participants attended the centre for an initial screening visit. The principal investigator or designee discussed with each participant the nature of the trial, its requirements and restrictions, in line with the participant information sheet previously given to the participant. Formal written consent was provided.

Before the baseline and week 26 assessments, participants were required to visit the center to collect an actiwatch, sleep diary, and urine sampling pack. Participants were required to wear the actiwatch and complete the sleep diary for the 7 nights, prior to the baseline and week 26 assessments, and to provide urine samples the night before and the morning of the baseline and week 26 assessments. Participants were asked to avoid alcohol and refrain from intake of ‘over the counter’ medications for 24 h and of caffeine for 18 h before both the baseline and week 26 assessments. Participants were contacted to remind them of the requirements prior to each assessment. On the morning of the baseline testing visit, participants were requested to eat their usual breakfast at least 1 h prior to arrival at the laboratory (but to avoid any caffeinated products) or to not have breakfast if they usually skipped breakfast. At the end of the baseline assessments, participants were provided with the first batch of capsules (3 bottles of 100 capsules each) and given a diary in which to record their daily consumption of capsules, along with any adverse events and concomitant medications (see Supplementary Figure S1 for schematic depiction of the study overview).

Participants also reported to the BPNRC during week 13 to collect the second batch of capsules (3 bottles of 100 capsules each) and to complete the Leeds Sleep Evaluation Questionnaire (LSEQ) and subjective awakening scales. Participants also brought with them their diary, which was replaced with a new diary to complete between week 13–26, and any remaining unused treatment capsules, so that a treatment compliance percentage could be calculated.

The week 26 testing assessment was identical to the baseline assessment in all aspects, apart from collecting the treatment and sleep diaries, all remaining treatments, completion of a treatment guess questionnaire, and finally a full debrief once all assessments were completed. During both baseline and week 26 visits, participants were also required to provide a 6 mL venous blood sample to determine red blood cell fatty acid profile.

### 2.7. Outcomes

#### 2.7.1. Subjective Measures

The LSEQ is a 10-item visual analog scale (VAS) specifically designed to measure changes in subjective sleep, following a pharmacological intervention [18]. The questionnaire measures aspects of sleep including Getting to Sleep, Quality of Sleep, Awakening from Sleep, and Behavior Following Sleep. The 10 items that made up the four sleep components were each presented on a 100 mm line with one end representing a negative and the other representing a positive response to the question. Higher scores on these scales represented more positive feelings of the respective items.

The VAS measured items related to participant’s subjective awakening state. Participants rated their current subjective state by making a mark on a 100 mm line with the end-points labelled “not at all” (left hand end) and “very much so” (right hand end). These scales included the following questions “how rested do you feel?”, “how energetic do you feel?”, “how relaxed do you feel?”, “how irritable do you feel?”, “how ready do you feel to perform”, and “have you had a good night’s sleep?”. Higher scores on these scales represent stronger feelings of the respective items.

#### 2.7.2. Biological Measures

Urine sampling commenced on the evening prior to the baseline and week 26 testing visits and comprised three separate samples—void at bedtime and the first and second voids of the following day (morning of the testing visit). If a participant needed to urinate during the night, then these voids were also collected in the same manner, as described below.

Urine was collected in a sterilized measuring cylinder. Void volume, time and date were recorded, before a 10 mL aliquot of urine was retained and refrigerated in a screw cap container, pre-labelled with the participant’s study details. The samples were taken to the laboratory at the baseline and week 26 testing visits, for further labelling and immediate storage at −80 °C for later analysis of the major melatonin metabolite 6-sulfatoxymelatonin (aMT6s), using radioimmunoassay [19].

Total excretion of aMT6s (ng) summed from all voids and the bedtime aMT6s (ng) values were calculated. Bedtime excretion of aMT6s specifically was also chosen to be analyzed independently from the total aMT6s, as a measure of melatonin production before sleeping, in an attempt to assess the effects of treatment on bedtime melatonin levels. This is because reduced evening melatonin production is associated with sleep disturbances [20], and urinary levels of aMT6s are seen to parallel those of melatonin in the blood, saliva, and urine [21].

#### 2.7.3. Objective Measures

Participants were instructed to complete sleep diaries to record time in and out of bed, and to wear actigraphy WGT3X-BT watches (ActiGraph LLC, Pensacola, FL, USA) on the non-dominant wrist for seven consecutive days and nights, both prior to commencing and before completing the 26-week supplementation period. The devices were small and lightweight and could detect body accelerations in the vertical, horizontal (right to left), and frontal (front and back) planes, at varying sample rates. The data from the watches were collected in 1-min epochs. Utilizing Actilife software (version 6.1, ActiGraph, Pensacola, FL, USA) and the Cole-Kripke algorithm [22], the following parameters could then be calculated:

- Sleep latency (The difference in minutes between In-Bedtime and sleep onset).
- Sleep efficiency (Number of sleep minutes divided by the total number of minutes the participant was in bed, i.e., the difference between the In-Bed and Out-Bedtime).
- Total sleep time (The total number of minutes scored as “asleep”).
- Total minutes in bed (The total number of minutes in bed both awake and asleep).
- Wake after sleep onset (The total number of minutes awake after sleep onset occurred).
- Number of awakenings (Total number of awakenings from the time spent in bed).
- Average awakening length (The average length, in minutes, of all awakening episodes).
- Sleep Fragmentation Index (The sum of the Movement Index—Total of scored awake minutes divided by Total time in bed in hours ×100 and the Fragmentation Index—Total of 1-min scored sleep bouts divided by the total number of sleep bouts of any length × 100)

### 2.8. Red Blood Cell Fatty Acid Measurements

Blood samples were collected via venepuncture into ethylenediaminetetraacetic acid vacutainers (6 mL) by trained phlebotomists. The samples were stored in an ice box, or at 5 °C, until they could be processed, which was within 8 h of collection. Blood was centrifuged at 2000 rpm (913× *g*) for 10 min at room temperature. The top layer of plasma was then removed and discarded. One ml of the red blood cell (RBC) pellet was collected, transferred to a 15 mL centrifuge tube and the volume was increased to 15 mL with phosphate-buffered saline (PBS). The mixture was inverted and centrifuged at 1200 rpm (350× *g*) for 10 min, at room temperature, with a low brake. The PBS was then removed and the washing process repeated for a second time. After the second wash, the RBC pellet was transferred into 1.5 mL microtubes and immediately frozen at −80 °C, prior to analysis.

RBC fatty acid composition was analyzed by gas chromatography [23]. The RBC pellet was washed twice with 5 mL of 0.9% sodium chloride (NaCl) and then total lipid was extracted using chloroform–methanol (2:1) containing 50 mg/L butylated hydroxytoluene as an antioxidant. The lipid phase was dried down under nitrogen, redissolved in a small volume of toluene, and then heated for 2 h at 50 °C with dry methanol containing 2% sulfuric acid. This procedure cleaved fatty acids from more complex lipids (e.g., membrane phospholipids) and simultaneously methylated them to produce fatty acid methyl esters (FAMEs). At the end of the reaction time, the sample was neutralized and FAMEs were extracted into hexane. FAMEs were concentrated and then separated on an Agilent 6890 gas chromatograph fitted with a 30-m long SGE BPX-70 fused silica capillary column. The split ratio was 25:1. The injector port temperature was 300 °C and helium was used as the carrier gas. The oven was held at 115 °C for 2 min, then increased at a rate of 10 °C per minute up to 200 °C, where it was held for 18.5 min. Oven temperature was then increased at a rate of 60 °C per minute to 245 °C, where it was held for 4 min. The flame ionization detector was held at 300 °C. FAMEs were identified by comparison with run times of authentic standards. Peak areas were calculated using the ChemStation software and each FAME was expressed as a weight % of the total. The *n*-3 index was calculated as % EPA + % DHA.

### 2.9. Compliance and Treatment Guess

As each participant was provided with 600 treatment capsules throughout the supplementation period, treatment compliance (%) could be calculated in order to measure adherence to the study protocol, with regards to appropriate consumption of the study treatments. Treatment compliance was calculated by comparing the number of capsules that were returned by each participant at the end of the study with the number of capsules that were to be returned.

Additionally, at the end of the study, all participants were provided with a treatment guess questionnaire and asked to choose between whether they had received an active or placebo treatment throughout the supplementation period, to verify the blinding procedure. Responses from the treatment guess questionnaire were analyzed via the chi-square test, comparing the number of correct and incorrect responses given by each treatment group.

### 2.10. Statistical Methods

Statistical analyses were performed with the IBM SPSS statistics software (version 25; IBM Corp, Armonk, NY, USA). Full data handling and cleaning procedures are described in the Supplementary Materials Section 2. Descriptive and comparison statistics (independent *t*-test, two-tailed, or chi-square test) of all baseline characteristics were based on all participants who were randomized and consumed at least one dose of treatment. All other analyses conducted were from the intention-to-treat (ITT) population. The general statistical approach selected to analyze the repeated measures data by treatment group was via linear mixed models (LMM) with treatment (DHA-rich, EPA-rich, placebo) and night (1–7) as factors in the objective sleep models and treatment (DHA-rich, EPA-rich, placebo), and visit (week 13 and week 26) in the subjective models. For each model that was run, the covariance matrix structure was chosen on the basis of structure that produced the lowest Schwarz’s Bayesian Criterion (BIC), an indication of the best fitting model for the data [24]. Changes within outcome variables during the treatment period were assessed via LMMs that adjusted for the respective baseline scores. Significant main or interaction effects of treatment (*p* < 0.050) were further investigated with Sidak-corrected comparisons to account for multiple group comparisons.

### 2.11. Ethics

This study was pre-registered via www.clinicaltrials.gov (NCT03559361) and conducted at the University of Northumbria, according to the guidelines of the Declaration of Helsinki (2013). Ethical approval for the trial was obtained from the University of Northumbria, Department of Psychology Ethics Committee (SUB023), and written informed consent was obtained from all participants. All paper study data were stored in a locked filing cabinet and electronic data on a secure network drive with access granted only to those working within the research center. The trial described in this manuscript was a sub-study of a larger study investigating the effects of the EPA- and DHA-rich oils on cognitive function (NCT02763514).

## 3. Results

The flow of participants through the study is summarized in Figure 1. The final analysis was conducted in 84 participants (n = 28 in the placebo group; n = 29 in the DHA-rich oil group; n = 27 in the EPA-rich oil group) for whom baseline and end of study data were available. Baseline characteristics of subjects are summarized in Table 1. No significant differences between the treatment groups were identified for any baseline demographics.

### 3.1. Compliance

For participants who completed the study, compliance was observed to be very good in all three groups (95.21% Placebo, 96.42% DHA-rich, 95.64% EPA-rich), with one-way ANOVA identifying no significant differences for compliance percentage by treatment group [*F* (2, 81) = 0.274, *p* = 0.761]. A chi-square test was also conducted on the responses to the treatment guess questionnaire that was completed at the end of the final visit and revealed no significant differences in participants’ ability to correctly identify whether they were administered an active or placebo treatment between the three groups [*χ^2^* (2) = 3.84, *p* = 0.147]. Analysis of RBC fatty acid profiles further supports the compliance data, with increases in EPA, DHA and *n*-3 index in both EPA and DHA groups (Table 3). This increase was more marked in the DHA than the EPA group (Table 3).

### 3.2. Mixed Models Analysis

Due to the number of possible interactions between the factors, only those that revealed significant main or interaction effects, including treatment, are reported.

#### 3.2.1. Objective Measures

**Figure 2:**
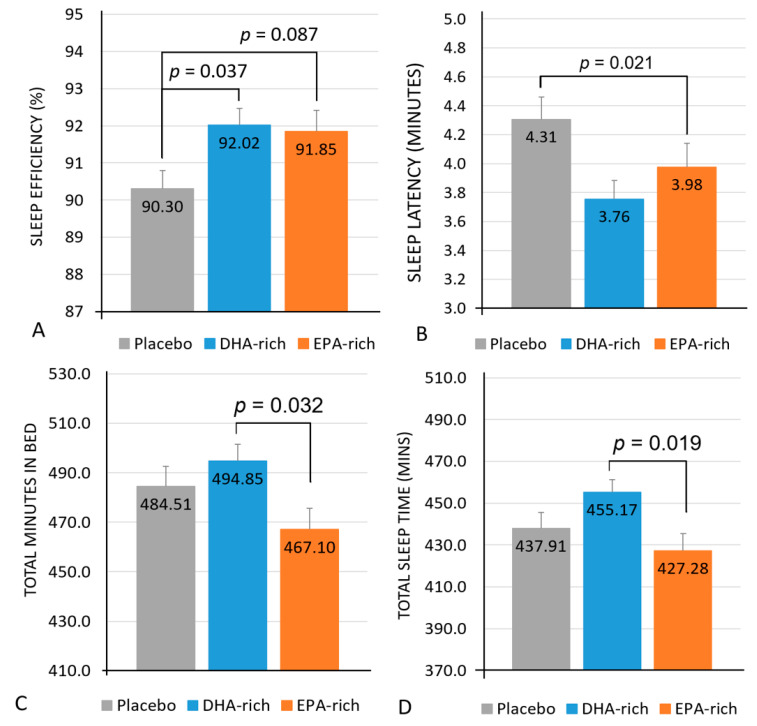
Estimated marginal means and standard error (SE) for post-treatment values of sleep efficiency (**A**), sleep latency (**B**), total minutes in bed (**C**), and total sleep time in minutes (**D**).

See Table 4 for a summary of all objective sleep results. A significant main effect of treatment for sleep efficiency was identified [*F* (2, 79.79) = 3.68, *p* = 0.030] with post-hoc comparisons identifying the DHA-rich group (92.02%; *p* = 0.037) as having significantly higher sleep efficiency, with a trend towards significantly higher sleep efficiency in the EPA-rich group (91.85%; *p* = 0.087) as compared to the placebo (90.30%) (Figure 2A).

Analysis identified a significant main effect of treatment for sleep latency [*F* (2, 322) = 3.68, *p* = 0.026] with post-hoc comparisons identifying the DHA-rich (3.76; *p* = 0.021) but not the EPA-rich (3.98; *p* = 0.276) group as showing significantly shorter sleep latency, as compared to placebo (4.31) (Figure 2B).

Analysis also identified a significant interaction between treatment and night for sleep latency [*F* (12, 322) = 2.28, *p* = 0.009], with post-hoc comparisons identifying the DHA-rich group (3.31) as having a significantly shorter latency period compared to both the placebo (6.43; *p* = 0.003) and the EPA-rich (5.80; *p* = 0.023) groups on night 1, and both the DHA-rich (3.36, *p* = 0.017) and EPA-rich (3.34, *p* = 0.021) groups as having a significantly shorter latency period compared to placebo (4.53) on night 6.

Analysis identified a significant main effect of treatment for total minutes in bed [*F* (2, 328) = 3.29, *p* = 0.039], with post-hoc comparisons identifying no significant differences between the active and placebo groups, but the DHA-rich group (484.51 min) spent significantly more time in bed than the EPA-rich group (467.10; *p* = 0.032) (Figure 2C).

A significant main effect of treatment was also identified for total sleep time [*F* (2, 323) = 4.06, *p* = 0.018] with post-hoc comparisons identifying no significant differences between the active and placebo groups but the DHA-rich group (455.17 min) spent significantly more time asleep than the EPA-rich group (427.28; *p* = 0.019) (Figure 2D).

Analysis also identified a significant interaction between treatment and night for the sleep fragmentation index [*F* (12, 227.64) = 1.90, *p* = 0.025], with post-hoc comparisons identifying the DHA-rich group (15.88; *p* = 0.003) as having significantly less sleep fragmentation compared to placebo (26.85) on night 2 only.

#### 3.2.2. Subjective Measures

**Figure 3:**
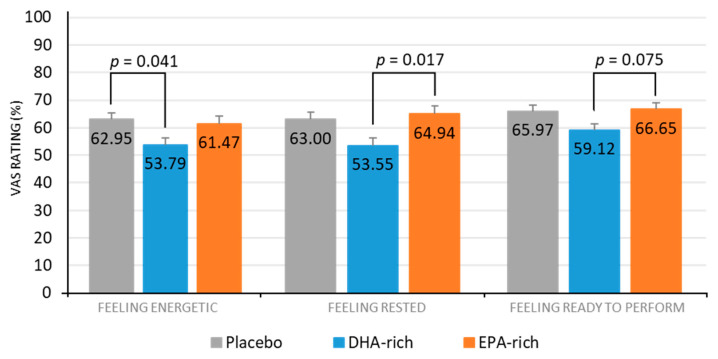
Estimated marginal means and standard error (SE) for post-treatment subjective ratings for feeling energetic, feeling rested, and feeling ready to perform, by treatment group.

See Table 5 for a full summary of subjective sleep results. A significant effect of treatment for feeling energetic was also identified [*F* (2, 79.35) = 3.545, *p* = 0.034], with post-hoc comparisons identifying the DHA-rich (53.79; *p* = 0.041) but not the EPA-rich (64.94; *p* = 0.970) group as feeling significantly less energetic, as compared to the placebo (62.47) (Figure 3).

A significant effect of treatment for feeling rested was identified [*F* (2, 76.42) = 4.71, *p* = 0.017], with post-hoc comparisons identifying no significant difference between the active and placebo groups, but the DHA-rich group (53.55) were significantly less rested than the EPA-rich group (64.94; *p* = 0.017) (Figure 3).

A significant effect of treatment for feeling ready to perform was identified [*F* (2, 84.12) = 3.211, *p* = 0.045], with post-hoc comparisons identifying no significant difference between the active and placebo groups but the DHA-rich group (59.12) showed a trend towards being significantly less ready to perform than the EPA-rich group (66.65; *p* = 0.075) (Figure 3).

A significant treatment by visit interaction for behavior, following waking, was observed [*F* (2, 77.01) = 5.03, *p* = 0.009]. However, post-hoc comparisons identified no significant differences between any of groups at either week 13 or 26. No other effects of treatment were observed for any other subjective measures.

#### 3.2.3. Biological Measures

No significant main effects of treatment were observed for urinary aMT6s (Table 6).

## 4. Discussion

The results from the current study show that supplementation with DHA-rich oil in healthy adults who do not habitually consume oily fish, resulted in a significant increase in sleep efficiency and a significant decrease in sleep latency compared to placebo. Interestingly, despite these improvements in the objective actigraphy sleep measures in the DHA-rich group, it was also found that this group reported feeling less rested compared to placebo, and less energetic and ready to perform than those given EPA-rich oil. A significant decrease in the sleep fragmentation index was also observed in the DHA-rich group, as compared to the placebo. However, the latter effect was found to only be evident during the second night of the seven nights recorded, and must be interpreted with caution. With regards to the EPA-rich oil, a trend towards a significant increase in sleep efficiency was identified in this group, as compared to the placebo. The EPA-rich oil also resulted in a significant decrease in both total time in bed and total sleep time, as compared to the DHA-rich group, although no significant differences were identified between either treatment group and placebo for these measures. Finally, no significant effects of treatment were identified for urinary aMT6s excretion.

The beneficial effects of DHA in increasing sleep efficiency and reducing sleep latency were consistent with previous animal models [25] and exploratory data from an intervention study in children [11], providing further evidence to support the beneficial role of DHA in sleep. Indeed, enzymatic transformation of serotonin to melatonin by aralkylamine N-acetyltransferase [26] was supported by DHA, via its positive effects on membrane fluidity [27] and serotonin levels in the prefrontal cortex [28], which might help modulate the transition between sleep and wakefulness [29]. Given the above, the null findings of treatment on urinary aMT6s might suggest that DHA affects sleep via mechanisms other than the melatonin synthesis pathway. However, it might also be the case that the period of urinary collection over a single night was simply not sensitive enough to identify an effect on aMT6s. Therefore, in order to better evaluate the relationship between *n*-3 PUFAs, melatonin and sleep, future research should consider either the use of 24/48 h urinary collection periods or the analysis of melatonin in blood, which allows for greater resolution and sensitivity [30].

The negative subjective ratings identified in the DHA-rich oil group were inconsistent with the actigraphy data. One potential explanation for this might be informed by investigations of patients suffering from insomnia. For instance, Feige et al. [31] explain how a major enigma of insomnia research constitutes the frequently noted discrepancy between the subjective experience of sleep (measured by sleep questionnaires) and the polysomnographic (PSG) findings. PSG studies often demonstrate that patients suffering from insomnia tend to underestimate their nocturnal sleep time [32,33], leading to terms such as ‘sleep state misperception’ for patients with a relatively normal sleep continuity and architecture, despite subjective complaints of disturbed sleep [34]. Due to the issues with objectively defining sleep parameters (e.g., sleep efficiency/latency [35]), focusing on the architecture of sleep might offer additional explanations for these conflicting data. For example, Feige et al. [32] showed that differences between subjectively and objectively measured wake times were correlated with the amount of REM sleep in insomnia patients, i.e., patients with higher amounts of REM sleep tended to report more minutes of subjective wakefulness. Further investigation using PSG would therefore provide valuable insights into the effects of *n*-3 PUFAs on the sleep architecture, in relation to the amounts of REM and non-REM sleep, which could then be evaluated alongside subjective effects.

Regarding the observed effects of EPA-rich oil on sleep, the differential pattern of results compared to placebo and the direct differences between the effects of each treatment do suggest specific roles of DHA and EPA in sleep. The shortened sleep times identified within the current study, following the EPA-rich oil as compared to the DHA-rich oil, might potentially be explained by the role of EPA inhibiting the formation of E_2_-series prostaglandins, which in turn inhibit the release of serotonin [36]. As serotonin promotes wakefulness and inhibits REM sleep [37], it might be that increased levels of circulating EPA indirectly upregulate the promotion of wakefulness, resulting in decreased sleep time. It should be noted that although participants in the EPA-rich oil group reported the shortest sleep times, this did not appear to lead to any reduction in the quality of sleep. In fact, a trend towards a significant increase in sleep efficiency, compared to placebo, was observed along with no increases in the time spent awake, number of awakenings, or decreased ratings of subjective sleep quality. This might potentially suggest that EPA is beneficial for regulating a healthy sleep cycle and could help protect against suboptimal sleep (i.e., too little or too much sleep), which is known to be detrimental for health [38,39].

The current study was the first to investigate the separate effects of DHA and EPA on sleep, in a sample of healthy, young adults, with a rigorous study protocol that collected both objective and subjective measurements of sleep. Additionally, the measurement of aMT6s offered the potential to gain insight into possible mechanisms underpinning the relationship between *n*-3 PUFAs and sleep. The study had good compliance, as confirmed by measuring the RBC EPA, DHA, and the *n*-3 index. However, the study is not without its limitations and several challenges were faced with regards to the collection of actigraphy data, as well as with the subjective recording of sleep/wake times. Issues with incomplete and even unusable actigraph data—as a result of improper use of the equipment—resulted in a reduced sample size in the actigraphy datasets, although this reduction is in line with missing data observed in previous actigraphy studies [11,40]. Furthermore, future research might wish to take body composition into account when recruiting participants. As overweight and obese individuals are seen to have increased inflammatory profiles [41,42] and are more likely to experience sleep disorders [43], this might be a factor that future trials could control for more strictly or take into consideration during data analysis. As the current study included participants with a BMI ≤ 35 kg/m^2^, it could be that a more conservative range of BMI should be used in future.

Overall, this study provides additional support for the beneficial role of *n*-3 PUFAs, particularly DHA, for sleep. These include an overall increase in sleep efficiency and a reduction in sleep latency, although these positive measures of increased sleep quality measured using actigraphy were not consistent with subjective ratings, following DHA-rich oil. Further investigations into the relationship between *n*-3 PUFAs and the serotonin/melatonin synthesis pathway and effects on sleep architecture are required. Nonetheless, as beneficial effects of sleep were identified following supplementation with *n*-3 PUFAs in healthy, young adults, these data help to provide additional evidence towards the role of *n*-3 PUFAs in facilitating healthy regulation of sleep.

## Figures and Tables

**Table 1:** Demographic information and baseline characteristics for the 84 participants who completed all aspects of the study. Means ± SD are given where appropriate. Baseline differences were assessed using separate one-way ANOVAs or chi-square tests; resulting *p* values from these analyses are presented.

Variable	Treatment	Mean	SD	*p*
*n* (Males/Females)	Placebo	8/20	-	
	DHA-rich	7/22	-	0.886
	EPA-rich	8/19	-	
% of EPA in RBC	Placebo	0.82	0.21	
	DHA-rich	0.89	0.28	0.169
	EPA-rich	1.05	0.67	
% of DHA in RBC	Placebo	4.87	0.94	
	DHA-rich	4.74	0.92	0.637
	EPA-rich	5.04	1.50	
*n*-3 index (EPA + DHA)	Placebo	5.69	1.01	
	DHA-rich	5.63	1.06	0.455
	EPA-rich	6.10	2.00	
Age (years)	Placebo	36.89	7.78	
	DHA-rich	37.41	7.28	0.768
	EPA-rich	35.89	8.73	
Systolic BP (mmHg)	Placebo	122.80	11.19	
	DHA-rich	120.21	13.04	0.699
	EPA-rich	120.50	13.43	
Diastolic BP (mmHg)	Placebo	81.00	8.12	
	DHA-rich	79.45	9.67	0.805
	EPA-rich	79.81	9.82	
Heart Rate (BPM)	Placebo	71.70	12.36	
	DHA-rich	69.50	11.38	0.439
	EPA-rich	73.43	10.42	
Weight (kg)	Placebo	73.92	17.45	
	DHA-rich	70.37	12.20	0.656
	EPA-rich	72.11	14.40	
Height (cm)	Placebo	168.83	9.85	
	DHA-rich	166.59	6.01	0.614
	EPA-rich	167.65	8.48	
BMI (kg/m^2^)	Placebo	25.76	4.59	
	DHA-rich	25.36	4.24	0.936
	EPA-rich	25.62	4.48	
Years in Education	Placebo	16.28	1.10	
	DHA-rich	15.56	1.74	0.239
	EPA-rich	15.96	1.61	
Fruit & Vegetable (portions per day)	Placebo	4.13	1.78	
	DHA-rich	4.48	2.21	0.359
	EPA-rich	4.11	1.93	
Alcohol (units per day)	Placebo	1.00	0.71	
	DHA-rich	1.27	1.05	0.157
	EPA-rich	1.23	0.85	

**Table 2:** Outcomes from the DHA food frequency questionnaire for the 84 participants who completed all aspects of the study. Group differences were assessed using separate one-way ANOVAs; resulting *p* values from these analyses are presented.

N-3 PUFA Food Source	Treatment	Mean	SD	*p*
Oily fish, servings per month ^a^	Placebo	1.43	1.45	
	DHA-rich	1.79	1.55	0.522
	EPA-rich	1.85	1.48	
Fish, servings per month ^b^	Placebo	2.07	1.54	
	DHA-rich	2.05	1.76	0.834
	EPA-rich	2.33	2.41	
Fish/shellfish, servings per month ^c^	Placebo	1.36	1.70	
	DHA-rich	1.90	1.81	0.402
	EPA-rich	1.39	1.50	
Liver, servings per month ^d^	Placebo	2.04	4.78	
	DHA-rich	0.78	1.84	0.381
	EPA-rich	1.93	4.12	
Egg yolks, servings per week	Placebo	4.11	3.62	
	DHA-rich	4.52	4.12	0.890
	EPA-rich	4.69	5.77	
Poultry, servings per week	Placebo	2.93	2.36	
	DHA-rich	3.53	2.59	0.534
	EPA-rich	2.87	2.44	

^a^ includes bluefish, blue fin tuna, cisco (smoked), herring, mackerel, pollock, sardines, salmon, whitefish; ^b^ includes bass, calamari, catfish, drumfish, flounder, grouper, hailbut, mussels, perch, redfish, rockfish, shark, snapper, sole, squid, swordfish, trout, tuna (canned 6 oz), whiting; ^c^ includes carp, clams, cod, crab, crayfish, fish patties/squares, fish sticks, haddock, lobster, mullet, oysters, pike, pompano, scallops, shrimp (14 medium), surgeon; and ^d^ includes chicken liver, turkey liver, or beef liver.

**Table 3:** Red blood cell EPA, DHA, and *n*-3 index for placebo, DHA-rich, and EPA-rich treatment groups. Data are mean ± SD at baseline, week 26, and change (from baseline).

Variable	Treatment	Baseline (*n* = 80)	Week 26 (*n* = 70)	Change * (*n* = 69)
% of EPA in RBC	Placebo	0.82 ± 0.21	0.80 ± 0.27	−0.03 ± 0.19
	DHA-rich	0.88 ± 0.28	2.16 ± 0.57	1.24 ± 0.57
	EPA-rich	1.03 ± 0.43	2.73 ± 1.02	1.68 ± 1.03
% of DHA in RBC	Placebo	4.82 ± 0.96	4.77 ± 0.82	0.03 ± 0.78
	DHA-rich	4.71 ± 0.91	7.69 ± 1.31	2.94 ± 1.42
	EPA-rich	5.04 ± 1.48	6.12 ± 0.95	1.08 ± 1.43
*n*-3 index (EPA + DHA)	Placebo	5.63 ± 1.03	5.57 ± 0.95	−0.00 ± 0.80
	DHA-rich	5.59 ± 1.06	9.85 ± 1.64	4.18 ± 1.69
	EPA-rich	6.07 ± 1.94	8.85 ± 1.60	2.75 ± 2.19

* Change values are only calculated for those participants who had data at both baseline and week 26.

**Table 4:** Objective sleep outcomes for the placebo, DHA-rich, and EPA-rich treatment groups. Post-dose estimated marginal means and standard error (SE) are presented with *F* and *p* values of the main effects from the linear mixed models.

Variable	Treatment		Post-Dose		Main Effects		
		n	Mean	SE		*F*	*p*
Latency (minutes)	Placebo	74	4.31 ^a^	0.21	Treatment	3.68	0.026
	DHA-rich		3.76 ^a^	0.26			
	EPA-rich		3.98	0.27	Treatment × Night	2.28	0.009
Efficiency (%)	Placebo	72	90.30 ^a,T^	0.50	Treatment	3.68	0.030
	DHA-rich		92.02 ^a^	0.49			
	EPA-rich		91.85 ^T^	0.57	Treatment × Night	1.47	0.138
Total Minutes in bed (minutes)	Placebo	74	484.51	8.13	Treatment	3.29	0.039
	DHA-rich		494.85 ^b^	6.63			
	EPA-rich		467.10 ^b^	8.55	Treatment × Night	0.851	0.598
Total Sleep Time (Minutes)	Placebo	73	437.91	7.56	Treatment	4.06	0.018
	DHA-rich		455.17 ^b^	6.18			
	EPA-rich		427.28 ^b^	8.08	Treatment × Night	1.20	0.281
Wake after Sleep Onset (minutes)	Placebo	72	42.02	2.42	Treatment	2.55	0.084
	DHA-rich		35.84	2.14			
	EPA-rich		34.77	2.74	Treatment × Night	1.29	0.225
Number of Awakenings	Placebo	74	17.50	0.99	Treatment	0.813	0.446
	DHA-rich		15.87	0.88			
	EPA-rich		16.20	1.08	Treatment × Night	1.19	0.289
Average Awakening Length (minutes)	Placebo	74	2.44	0.11	Treatment	0.576	0.564
	DHA-rich		2.29	0.09			
	EPA-rich		2.38	0.12	Treatment × Night	1.50	0.126
Sleep Fragmentation Index	Placebo	74	22.89	1.28	Treatment	0.802	0.451
	DHA-rich		20.80	1.11			
	EPA-rich		22.22	1.38	Treatment × Night	1.90	0.036

^a^ = significant difference between active and placebo groups, *p* < 0.050; ^b^ = significant difference between the active treatment groups, *p* < 0.050; ^T^ = trend towards a significant difference between active and placebo groups, *p* < 0.100.

**Table 5:** Subjective sleep outcomes for the placebo, DHA-rich, and EPA-rich treatment groups. Estimated marginal means and standard error (SE) for week 13 and week 26 are presented with F and *p* values of the main effects from the linear mixed models.

Variable	Treatment		Week 13		Week 26		Main Effects		
		n	Mean	SE	Mean	SE		*F*	*p*
Getting to Sleep (0–300)	Placebo	86	182.49	6.69	170.63	6.69	Treatment	0.243	0.785
	DHA-rich		177.13	7.28	177.05	6.56			
	EPA-rich		176.04	7.09	167.88	6.98	Treatment × Visit	0.557	0.575
Quality of Sleep (0–200)	Placebo	86	118.12	6.65	112.38	6.74	Treatment	0.438	0.647
	DHA-rich		118.47	7.19	118.64	6.53			
	EPA-rich		109.22	7.02	112.26	6.92	Treatment × Visit	0.392	0.677
Awake Following Sleep (0–200)	Placebo	86	107.23	6.01	113.52	6.12	Treatment	0.518	0.598
	DHA-rich		118.80	6.62	115.34	5.91			
	EPA-rich		112.80	6.37	113.05	6.27	Treatment × Visit	0.379	0.686
Behaviour Following Wakening (0–300)	Placebo	86	191.09	7.68	180.02	7.79	Treatment	0.814	0.447
	DHA-rich		181.98	8.38	165.39	7.57			
	EPA-rich		169.66	8.20	188.93	8.09	Treatment × Visit	5.03	0.009
Rested (%)	Placebo	86	66.21 ^a^	3.67	59.80	3.74	Treatment	4.71	0.012
	DHA-rich		56.44 ^a^	4.06	50.65	3.60			
	EPA-rich		68.79	3.91	61.09	3.82	Treatment × Visit	0.034	0.966
Energetic (%)	Placebo	86	65.69 ^a^	3.21	60.20	3.26	Treatment	3.55	0.034
	DHA-rich		56.35 ^a^	3.56	51.23	3.16			
	EPA-rich		60.42	3.42	62.51	3.37	Treatment × Visit	1.05	0.354
Relaxed (%)	Placebo	86	64.87	3.21	65.82	3.26	Treatment	1.37	0.260
	DHA-rich		61.12	3.49	58.81	3.14			
	EPA-rich		65.60	3.40	65.47	3.35	Treatment × Visit	0.191	0.827
Irritable (%)	Placebo	86	26.74	3.70	27.70	3.77	Treatment	1.46	0.238
	DHA-rich		31.95	4.09	35.10	3.64			
	EPA-rich		28.70	3.92	27.25	3.85	Treatment × Visit	0.196	0.822
Ready to Perform (%)	Placebo	86	65.70	2.81	66.23	2.86	Treatment	3.21	0.045
	DHA-rich		61.56 ^b^	3.08	56.68	2.76			
	EPA-rich		66.88 ^b^	2.98	66.43	2.92	Treatment × Visit	0.668	0.515
Good Night’s Sleep (%)	Placebo	86	65.88	4.30	59.53	4.38	Treatment	1.61	0.205
	DHA-rich		63.06	4.72	50.85	4.23			
	EPA-rich		68.37	4.54	62.69	4.45	Treatment × Visit	0.392	0.677

^a^ = significant difference between active and placebo groups, *p* < 0.050; ^b^ = significant difference between the active treatment groups, *p* < 0.050.

**Table 6:** Urinary aMT6s for placebo, DHA-rich, and EPA-rich treatment groups. Post-treatment estimated marginal means and standard error (SE) are presented with the F and *p* values of the main effects from the linear mixed models.

Variable	Treatment		Post-Treatment		Main Effects		
		n	Mean	SE		*F*	*p*
Total aMT6s (ng)	Placebo	67	15,289.27	1,267.50			
	DHA-rich		15,335.89	1,267.88	Treatment	0.558	0.575
	EPA-rich		13,585.56	1,346.06			
Bedtime aMT6s (ng)	Placebo	60	563.98	120.67			
	DHA-rich		468.62	123.42	Treatment	2.12	0.130
	EPA-rich		805.34	117.08			

## Data Availability

Data outputs for this study are available by contacting the corresponding author.

## Supplementary Materials

The following are available online at https://www.mdpi.com/2072-6643/13/1/248/s1. Figure S1: Schematic showing the study progression from enrolment to completion across the 26 weeks. Actigraphy recordings were taken for the seven days and nights prior to the baseline; week 26 testing visits and urinary aMT6s samples were collected the night prior to and morning of the baseline and week 26 testing visits. LSEQ—Leeds Sleep Evaluation Questionnaire.
