# Supplementary material for: Differential Effects of DHA- and EPA-Rich Oils on Sleep in Healthy Young Adults: A Randomized Controlled Trial
Source: Nutrients. 2021 Jan 16;13(1):248. doi: 10.3390/nu13010248 (PMC7830450; doi:10.3390/nu13010248)
Supplement: Supplementary file 1 [file nutrients-13-00248-s001.pdf]

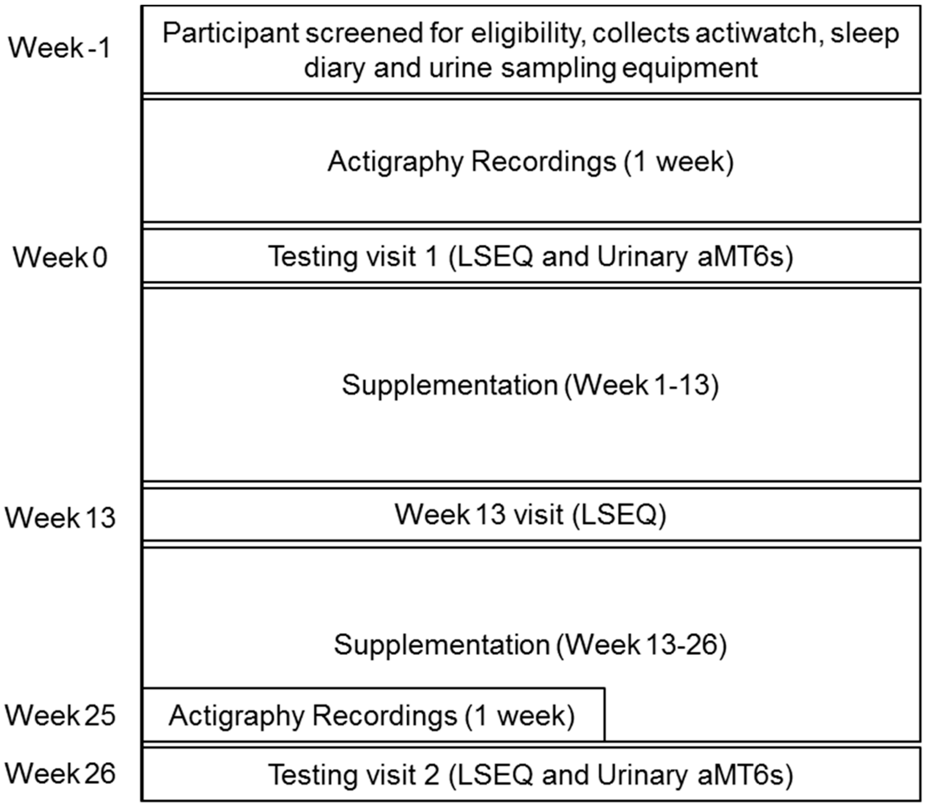

Figure 1. Schematic showing the study progression from enrollment to completion across the 26 weeks. Actigraphy recordings were taken for the seven days and nights prior to the baseline and week 26 testing visits and urinary aMT6s samples were collected the night prior to and morning of the baseline and week 26 testing visits. LSEQ, Leeds Sleep Evaluation Questionnaire.

Statistical Methods

1. Data Cleaning Procedures

1.1. General

Before each analysis was conducted the data sets were cleaned following the same procedures. These procedures included removing anomalous results and outliers from the raw data. All analyses were completed using SPSS (version 25) and box plots were generated for each outcome variable to identify potential outliers. These boxplots present five sample statistics - the minimum, the lower quartile, the median, the upper quartile and the maximum. SPSS has a two stage flagging process. Values which are between one and a half and three box lengths from either end are denoted by open circles and are interpreted as outliers. Values which are more than three box lengths from either end of the box are denoted by asterisks and interpreted as extreme values. Once any identified outliers had been removed, residual values were calculated and histograms produced to view the spread and distribution of the data. If any values were seen to be separate from the spread and distribution from the dataset then these values were also removed. Once these processes had been completed for each outcome variable the analysis commenced.

1.2. Actigraphy

Initially, automatically detected sleep/wake times were used to score the actigraphy data using the Cole-Kripke (ActiGraph) algorithm [1]. This algorithm automatically detects sleep/wake periods based on activity data from the present epoch, the preceding epoch and the following epochs. This algorithm is considered appropriate for use with adult populations as it was developed using participants ranging from 35 to 65 years of age. The Cole-Kripke algorithm was validated in adults wearing a Motionlogger Actigraph (Ambulatory Monitoring, Inc.). In order to match the output of

the more sensitive WGT3X-BT device with the Motionlogger Actigraph and AMA-32, ActiGraph adapted the original Cole-Kripke algorithms to the ActiGraph devices by performing a side-by-side test using devices from both companies worn together [2, 3].

However, whilst following the automatic detection of sleep/wake times several issues arose. It became apparent that the automatically identified sleep/wake times did not always compare with the data recorded in the participants sleep diaries; it appeared to often overestimate or underestimate sleep periods and failed to detect any useable sleep periods in 1/3rd of the overall dataset, resulting in a significantly diminished amount of data. These issues have been reported previously in the literature including inability of sleep algorithms to accurately detect sleep/wake times [4] and their tendency to overestimate/underestimate sleep/wake periods [5]. As a result, alternative methods can be employed to detect sleep/wake times, including the use of recorded sleep diaries or visually inspecting the data [4, 6-8]. Consequently, it was decided that to avoid the loss of a significant quantity of data, manual detection of the sleep/wake times was required. This involved visually inspecting the data for decreases and increases in activity, further directed by the participant's recorded in/out of bed times in their sleep diaries. This process was completed whilst researchers were still blind to treatment. Once the sleep/wake times had been manually entered for each participant during each night the same Cole-Kripke (ActiGraph) algorithm described previously was then used [1] to generate the sleep outcomes.

2. Linear Mixed Models

2.1. Actigraphy

Actigraphy data collected from the Actigraph sleep watches seven nights prior to the week 26 assessment were analysed with the seven night's prior to the baseline assessment acting as a covariate. Actigraphy data included sleep efficiency, sleep latency, total sleep time, total minutes in bed, wake after sleep onset, number of awakenings, average awakening length and sleep fragmentation index. Data were analysed using linear mixed models in SPSS (version 25) with the covariance structure being chosen from the model with the lowest Schwarz's Bayesian Criterion (BIC) indicating the best fitting model for the data. For the sleep latency, total minutes in bed and total sleep time models the identity covariance matrix was used whilst the sleep efficiency, wake after sleep onset, number of awakenings, average awakening length and sleep fragmentation index an autoregressive (first order) covariance matrix was used.

Fixed factors appearing in all actigraphy models were; treatment (DHA-rich, EPA-rich, Placebo) and night (1-7). Subject was also added into all models as a random factor and respective baseline values were entered into each model as a covariate.

2.2. LSEQ

The LSEQ data consisted of the individual summed scores on the items that comprised the quality of sleep, awake following sleep, behaviour following waking and getting to sleep factors. The data were analysed using the same linear mixed model procedure described above with all models using an identity covariance matrix. The fixed factors appearing in all models were; treatment (DHA-rich, EPA-rich, Placebo) and visit (week 13 and week 26). Subject was also added into all models as a random factor and respective baseline values were entered into each model as a covariate.

2.3. Awakening VAS

The awakening VAS data were analysed using the same linear mixed model procedure described above with all models using an identity covariance matrix. The fixed factors appearing in all models were; treatment (DHA-rich, EPA-rich, Placebo) and visit (week 13 and week 26). Subject was also added into all models as a random factor and respective baseline values were entered into each model as a covariate.

2.4. Urinary aMT6s

The urinary aMT6s data consisted of the total excretion of aMT6s (ng) summed from all voids and the bedtime aMT6s (ng) values. All urinary aMT6s data was analysed using the linear mixed

models procedure outlined above. The only fixed factor appearing in the model was treatment (DHA-rich, EPA-rich, Placebo) with respective baseline values entered as a covariate.

References

1. Cole, R.J., et al., Automatic Sleep/Wake Identification From Wrist Activity. *Sleep*, 1992. 15(5): p. 461-469.
2. ActiGraph Software Department, Actilife 6 user's manual. 2012: Actigraph LLC.
3. Quante, M., et al., Actigraphy-based sleep estimation in adolescents and adults: a comparison with polysomnography using two scoring algorithms. *Nat Sci Sleep*, 2018. 10: p. 13-20.
4. Chow, C.M., et al., Defining the rest interval associated with the main sleep period in actigraph scoring. *Nat Sci Sleep*, 2016. 8: p. 321-328.
5. Ancoli-Israel, S., et al., The role of actigraphy in the study of sleep and circadian rhythms. *Sleep*, 2003. 26(3): p. 342-92.
6. Lockley, S.W., D.J. Skene, and J. Arendt, Comparison between subjective and actigraphic measurement of sleep and sleep rhythms. *J Sleep Res*, 1999. 8(3): p. 175-83.
7. Paquet, J., A. Kawinska, and J. Carrier, Wake detection capacity of actigraphy during sleep. *Sleep*, 2007. 30(10): p. 1362-9.
8. Tétreault, É., et al., Actigraphy data in pediatric research: the role of sleep diaries. *Sleep Med*, 2018. 47: p. 86-92.
